# The complete genome sequence of the acarbose producer *Actinoplanes *sp. SE50/110

**DOI:** 10.1186/1471-2164-13-112

**Published:** 2012-03-23

**Authors:** Patrick Schwientek, Rafael Szczepanowski, Christian Rückert, Jörn Kalinowski, Andreas Klein, Klaus Selber, Udo F Wehmeier, Jens Stoye, Alfred Pühler

**Affiliations:** 1 Senior research group in Genome Research of Industrial Microorganisms, Center for Biotechnology, Bielefeld University, Universitätsstraße 27, 33615 Bielefeld, Germany; 2 Genome Informatics Research Group, Faculty of Technology, Bielefeld University, Universitätsstraße 25, 33615 Bielefeld, Germany; 3 Institute for Genome Research and Systems Biology, Center for Biotechnology, Bielefeld University, Universitätsstraße 27, 33615 Bielefeld, Germany; 4 Bayer HealthCare AG, Friedrich-Ebert-Str. 475, 42117 Wuppertal, Germany; 5 Bayer Technology Services GmbH, Friedrich-Ebert-Str. 475, 42117 Wuppertal, Germany; 6 Bergische Universität Wuppertal, Sportsmedicine, Pauluskirchstraße 7, 42285 Wuppertal, Germany; 7 Center for Biotechnology, Bielefeld University, Universitätsstraße 27, 33615 Bielefeld, Germany

**Keywords:** Genomics, Actinomycetes, Actinoplanes, Complete genome sequence, Acarbose, AICE

## Abstract

**Background:**

*Actinoplanes *sp. SE50/110 is known as the wild type producer of the alpha-glucosidase inhibitor acarbose, a potent drug used worldwide in the treatment of type-2 diabetes mellitus. As the incidence of diabetes is rapidly rising worldwide, an ever increasing demand for diabetes drugs, such as acarbose, needs to be anticipated. Consequently, derived *Actinoplanes *strains with increased acarbose yields are being used in large scale industrial batch fermentation since 1990 and were continuously optimized by conventional mutagenesis and screening experiments. This strategy reached its limits and is generally superseded by modern genetic engineering approaches. As a prerequisite for targeted genetic modifications, the complete genome sequence of the organism has to be known.

**Results:**

Here, we present the complete genome sequence of *Actinoplanes *sp. SE50/110 [GenBank:CP003170], the first publicly available genome of the genus *Actinoplanes*, comprising various producers of pharmaceutically and economically important secondary metabolites. The genome features a high mean G + C content of 71.32% and consists of one circular chromosome with a size of 9,239,851 bp hosting 8,270 predicted protein coding sequences. Phylogenetic analysis of the core genome revealed a rather distant relation to other sequenced species of the family Micromonosporaceae whereas *Actinoplanes utahensis *was found to be the closest species based on 16S rRNA gene sequence comparison. Besides the already published acarbose biosynthetic gene cluster sequence, several new non-ribosomal peptide synthetase-, polyketide synthase- and hybrid-clusters were identified on the *Actinoplanes *genome. Another key feature of the genome represents the discovery of a functional actinomycete integrative and conjugative element.

**Conclusions:**

The complete genome sequence of *Actinoplanes *sp. SE50/110 marks an important step towards the rational genetic optimization of the acarbose production. In this regard, the identified actinomycete integrative and conjugative element could play a central role by providing the basis for the development of a genetic transformation system for *Actinoplanes *sp. SE50/110 and other *Actinoplanes *spp. Furthermore, the identified non-ribosomal peptide synthetase- and polyketide synthase-clusters potentially encode new antibiotics and/or other bioactive compounds, which might be of pharmacologic interest.

## Background

*Actinoplanes *spp. are Gram-positive aerobic bacteria growing in thin hyphae very similar to fungal mycelium [1]. Genus-specific are the formation of characteristic sporangia bearing motile spores as well as the rare cell wall components meso-2,6-diaminopimelic acid, L,L-2,6-diaminopimelic acid and/or hydroxy-diaminopimelic acid and glycine [1-4]. Phylogenetically, the genus *Actinoplanes *is a member of the family Micromonosporaceae, order Actinomycetales belonging to the broad class of Actinobacteria, which feature G + C-rich genomes that are difficult to sequence [5,6].

*Actinoplanes *spp. are known for producing a variety of pharmaceutically relevant substances such as antibacterial [7-9], antifungal [10] and antineoplastic agents [11]. Other secondary metabolites were found to possess inhibitory effects on mammalian intestinal glycosidases, making them especially suitable for pharmaceutical applications [12-15]. In particular, the pseudotetrasaccharide acarbose, a potent α-glucosidase inhibitor, is used worldwide in the treatment of type-2 diabetes mellitus (non-insulin-dependent). As the prevalence of type-2 diabetes is rapidly rising worldwide [16] an ever increasing demand for acarbose and other diabetes drugs has to be anticipated.

Starting in 1990, the industrial production of acarbose is performed using improved derivatives of the wild-type strain *Actinoplanes *sp. SE50 (ATCC 31042; CBS 961.70) in a large-scale fermentation process [12,17]. Since that time, laborious conventional mutagenesis and screening experiments were conducted by the producing company Bayer AG in order to develop strains with increased acarbose yield. However, the conventional strategy, although very successful [18], seems to have reached its limits and is generally superseded by modern genetic engineering approaches [19]. As a prerequisite for targeted genetic modifications, the preferably complete genome sequence of the organism has to be known. Here, a natural variant representing a first overproducer of acarbose, *Actinoplanes *sp. SE50/110 (ATCC 31044; CBS 674.73), was selected for whole genome shotgun sequencing because of its publicity in the scientific literature [17,20-22] and its elevated, well measureable acarbose production of up to 1 g/l [23]. Most notably, the acarbose biosynthesis gene cluster has already been identified [17,21,24-26] and sequenced [GenBank:Y18523.4] in this strain. For most of the identified genes in the cluster, functional protein assignment has been accomplished [17,21,22,24-27], presenting a fairly complete picture of the acarbose biosynthesis pathway in *Actinoplanes *sp. SE50/110, reviewed in [17,28,29]. However, scarcely anything is known about the remaining genome sequence and its influence on acarbose production efficiency through e.g. nutrient uptake mechanisms or competitive secondary metabolite gene clusters.

We have previously reported on the obstacles of high-throughput next generation sequencing for the *Actinoplanes *sp. SE50/110 genome sequence [30], which was carried out at the Center for Biotechnology, Bielefeld University, Germany. Having extensive experience in sequencing microbial genomes with classical Sanger methods [31-33], 454 next generation pyrosequencing technology [34,35] and especially with high G + C content genomes [36-39], we were able to identify the causes for the initial *Actinoplanes *sp. SE50/110 sequencing run to result in an unusually high number of contiguous sequences (contigs). It was found that a large number of stable secondary structures containing high G + C contents were responsible for the inability of the emulsion PCR step of the 454 library preparation protocol to amplify these regions. This led to their absence in the Genome Sequencer FLX output and ultimately to the missing sequences between the contigs established by the Newbler assembly software. Fortunately, these problems could be solved in a second (whole-genome shotgun) run by adding a trehalose-containing emulsion PCR additive, and by increasing the read length [30]. Based on this draft genome sequence, we now present the scaffolding strategy for the remaining contigs and report on the successful gap closure procedure that led to the complete finishing of the *Actinoplanes *sp. SE50/110 genome sequence. Furthermore, results from gene finding and genome annotation are presented, revealing compelling insights into the metabolic potential of the acarbose producer.

## Results and discussion

### High-throughput pyrosequencing and annotation of the *Actinoplanes *sp. SE50/110 genome

The complete genome determination of the arcabose producing wild-type strain *Actinoplanes *sp. SE50/110 was accomplished by combining the sequencing data generated by paired end (PE) and whole-genome shotgun pyrosequencing strategies [30]. Utilizing the Newbler software (454 Life Sciences), the combined assembly of both runs resulted in a draft genome comprising 600 contigs (476 contigs ≥ 500 bases) and 9,153,529 bases assembled from 1,968,468 reads.

**Figure 1:**
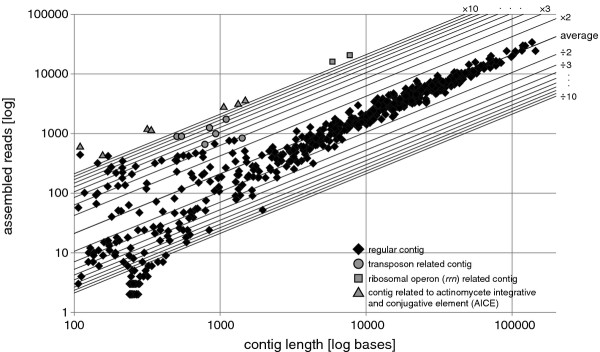
**Scatter plot of 600 *Actinoplanes *sp. SE50/110 contigs resulting from automatic combined assembly of the paired end and whole genome shotgun pyrosequencing runs**. The average number of reads per base is 43.88 and is depicted in the plot by the central diagonal line marked with 'average'. Additional lines indicate the factor of over- and underrepresentation of reads per base up to a factor of 10 and 1/10 fold, respectively. The axes represent logarithmic scales.

The contigs of the draft genome were analyzed for over- or underrepresentation in read coverage by means of a scatter plot to identify repeats, putative plasmids or contaminations (Figure 1). While most of the large contigs show an average coverage with reads, several contigs were found to be clearly overrepresented and are of special interest as discussed later. However, the majority of the unusual high and low covered contigs are of very short length, representing short repetitive elements (overrepresented) and contigs containing only few reads of low quality (underrepresented). These findings indicate clean sequencing runs without contaminations.

**Figure 2:**
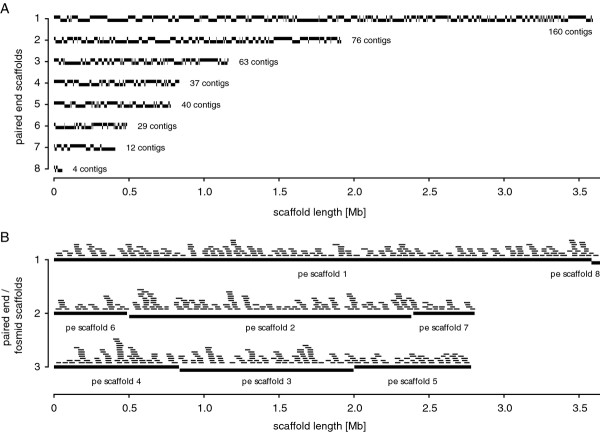
**Scaffolds of the *Actinoplanes *sp. SE50/110 genome**. **(A) **The eight paired end (PE) scaffolds resulting from Newbler assembly of all paired end and whole genome shotgun reads are shown. Every second contig is visualized in a slightly displaced manner to show contig boundaries. **(B) **The three scaffolds resulting from terminal insert sequencing of fosmid (FO) clones and subsequent mapping on the PE scaffolds are presented. All overlapping sequences of the 609 mapped fosmid clones are shown on top of the PE/FO scaffolds.

Based on PE information, 8 scaffolds were constructed using 421 contigs with an estimated total length of 9,189,316 bases (Figure 2A). These PE scaffolds were used to successfully map terminal insert sequences of 609 fosmid clones randomly selected from a previously constructed fosmid library (insert size of ~37 kb). The mapping results validated the PE scaffold assemblies and allowed the further assembly of the original 8 paired end scaffolds into 3 PE/fosmid (PE/FO) scaffolds due to bridging fosmid reads (Figure 2B).

**Figure 3:**
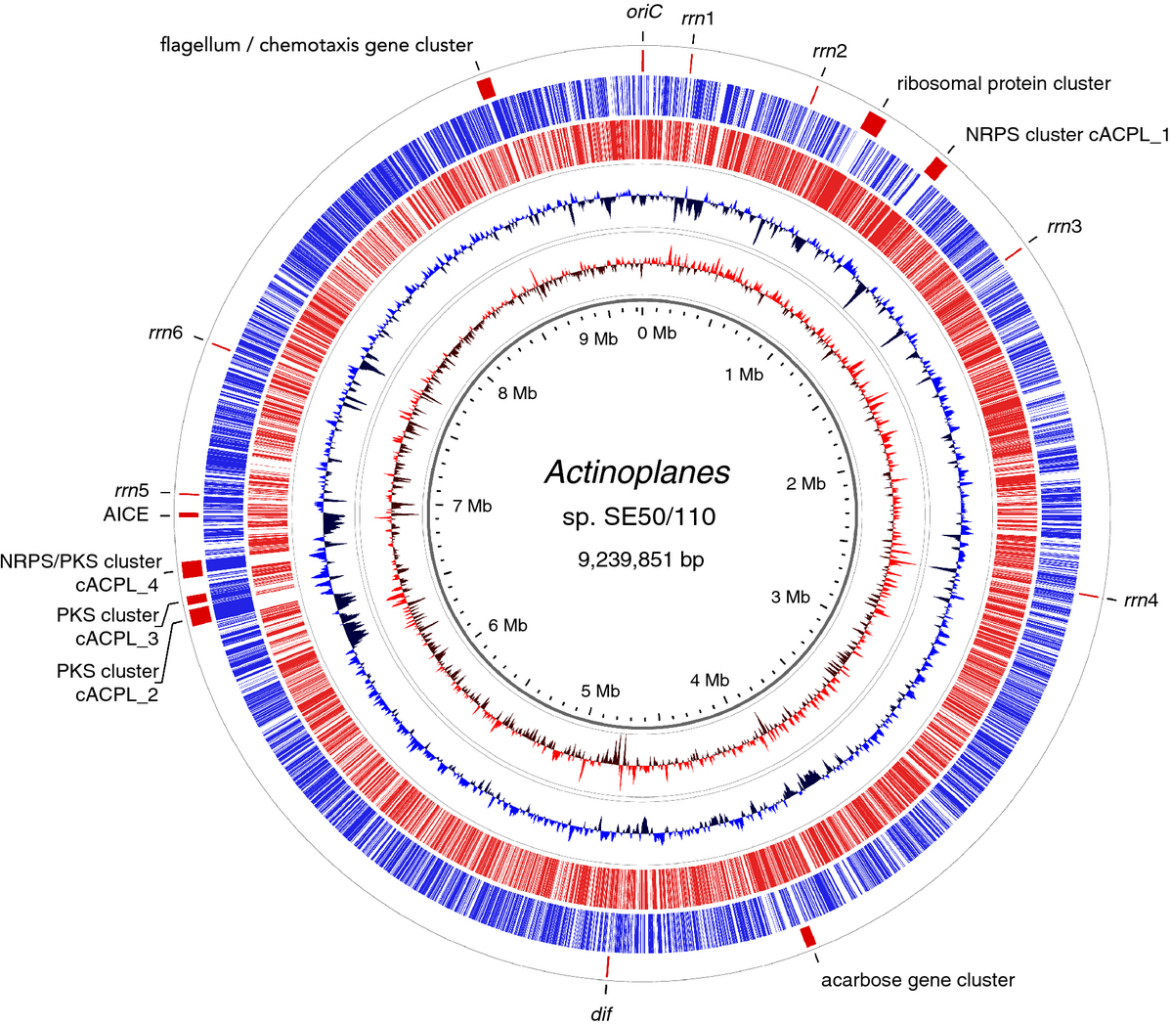
**Plot of the complete *Actinoplanes *sp. SE50/110 genome**. The genome consists of 9,239,851 base pairs and 8,270 predicted coding sequences. The circles represent from the inside: 1, scale in million base pairs; 2, GC skew; 3, G + C content (blue above- and black below genome average); 4, genes in backward direction; 5, genes in forward direction; 6, gene clusters and other sites of special interest. Abbrevations were used as follows: *oriC*, origin of replication; *dif*, chromosomal terminus region; *rrn*, ribosomal operon; NRPS, non-ribosomal peptide synthetase; PKS, polyketide synthase; AICE, actinomycete integrative and conjugative element.

Gap closure between the remaining contigs was carried out by fosmid walking (746 reads) and genomic PCR technology (236 reads) in cases were no fosmid was spanning the target region. Genomic PCR technology was also used to determine the order and orientation of the remaining 3 PE/FO scaffolds. The finishing procedure was manually performed using the Consed software [40] and resulted in the final assembly of a complete single circular chromosome of 9,239,851 bp with an average G + C content of 71.36% (Figure 3). According to genome project standards [41], the finished *Actinoplanes *sp. SE50/110 genome meets the gold standard criteria for high quality next generation sequencing projects. The general properties of the finished genome are summarized in Table 1.

**Table 1:** Features of the *Actinoplanes *sp. SE50/110 genome

Feature	Chromosome
Total size (bp)	9,239,851

G + C content (%)	71.32

No. of protein-coding sequences	8,270

No. of orphans	973

Coding density (%)	89.31

Average gene length (bp)	985

No. of rRNAs	6 × 16S-23S-5S

No. of tRNAs	99

Utilizing the prokaryotic gene finders Prodigal [42] and Gismo [43] in conjunction with the GenDB annotation pipeline [44], a total of 8,270 protein-coding sequences (CDS) were determined on the *Actinoplanes *sp. SE50/110 genome (Figure 3). These include 4,999 genes (60.5%) with an associated functional COG category [45], 2,202 genes (26.6%) with a fully qualified EC-number [46] and 973 orphan genes (11.8%) with neither annotation nor any similar sequence in public databases using BLASTP search with an e-value cutoff of 0.1. In total, the amount of protein coding genes (coding density) covers 90.11% of the genome sequence with a significant difference of 4% in G + C content between non-coding (67.74%) and coding (71.78%) regions.

The complete annotated genome sequence was deposited at the National Center for Biotechnology Information (NCBI) [GenBank:CP003170].

### General features of the *Actinoplanes *sp. SE50/110 genome

The origin of replication (*oriC*) was identified as a 1266 nt intergenic region between the two genes *dnaA *and *dnaN*, coding for the bacterial chromosome replication initiator protein and the β-sliding clamp of the DNA polymerase III, respectively. The *oriC *harbors 24 occurrences of the conserved DnaA box [TT(G/A)TCCACa], showing remarkable similarity to the *oriC *of *Streptomyces coelicolor *[47]. Almost directly opposite of the *oriC*, a putative *dif *site was found. Its 28 nt sequence 5'-CAGGTCGATAATGTATATTATGTCAACT-3' is in good accordance with actinobacterial *dif *sites and shows highest similarity (only 4 mismatches) to that of *Frankia alni *[48]. In addition to the identified *oriC *and *dif *sites, the calculated G/C skew [(G-C)/(G + C)] suggests two replichores composing the circular *Actinoplanes *sp. SE50/110 genome (Figure 3).

In accordance with previous findings [49], six ribosomal RNA (*rrn*) operons were identified on the genome in the typical 16S-23S-5S order along with 99 tRNA genes determined by the tRNAscan-SE software [50]. The six individual *rrn *operons were previously assembled into one operon ranging across seven contigs with a more than ten-fold overrepresentation (Figure 1). This overrepresentation might be explained by the operon's remarkably low G + C content of 57.20% in comparison to the genome average of 71.36%, which is typical for actinomycetes [49]. The low G + C content in this area may have introduced an amplification bias in favor of the *rrn *operon during the library preparation and thus, result in an overrepresentation of reads for this genomic region. To account for single nucleotide polymorphisms (SNPs) and variable regions between ribosomal genes, all six *rrn *operons were individually re-sequenced by fosmid walking. The *rrn *operons are located on the leading strands, four on the right and two on the left replichore. Interestingly, they reside in the upper half of the genome, together with a ~40 kb gene cluster hosting more than 30 ribosomal proteins (Figure 3). Other overrepresented large contigs were identified as transposase genes or transposon related elements (Figure 1).

Approximately 500 kb upstream of the *oriC *site, a flagellum gene cluster was found. Its expression in spores is one of the characteristics discriminating the genus *Actinoplanes *from other related genera [1,4]. The cluster consists of ~50 genes spanning 45 kb. Besides flagellum associated proteins, the cluster also contains genes coding for chemotaxis related proteins.

**Figure 4:**
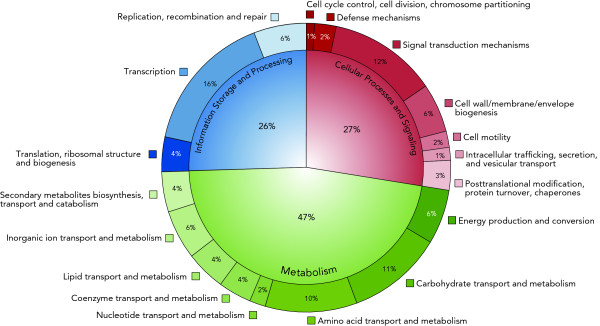
**Functional classifications of the *Actinoplanes *sp. SE50/110 protein coding sequences (CDS)**. The diagram represents the CDS that were categorized according their cluster of orthologous groups of proteins (COG) number [45,51]. All depicted percentages refer to the distribution of 4999 annotated CDS (100%) across all COG categories to which at least 10 CDS were found. Sequences with an unknown or poorly characterized function were excluded from the analysis. These excluded CDS were found to be rather randomly distributed across the genome. The outer ring contains specialized subclasses of the three main functional categories "Cellular Processes and Signaling", "Metabolism", and "Information Storage and Processing", located at the center.

Bioinformatic classification of 4,999 CDS with an annotated COG-category revealed a strong emphasis (47%) on enzymes related to metabolism (Figure 4). In particular, *Actinoplanes *sp. SE50/110 features an emphasis on amino acid (10%) and carbohydrate metabolism (11%), which is in good accordance with the identification of at least 29 ABC-like carbon substrate importer complexes. Furthermore, 16% of the COG-classified CDS code for proteins involved in transcriptional processes, which suggests a high level of gene-expression regulation. This is especially relevant for the ongoing search for a regulatory element or - network controlling the expression of the acarbose biosynthetic gene cluster. Interestingly, the great proportion of transcriptional regulators is accompanied by a similar high percentage of proteins involved in signal transduction mechanisms (12%), which suggests a close connection between extracellular nutrient sensing and transcriptional regulation of uptake systems and degradation pathways. Comparatively, an analogous analysis of 4,431 annotated CDS from *Streptomyces coelicolor *revealed an even higher percentage of genes coding for enzymes related to metabolism (55%), a minor portion dedicated to signal transduction mechanisms (7%), and a highly similar amount (16%) of proteins involved in transcriptional processes. Finally, the genome of *Actinoplanes *sp. SE50/110 reveals a striking focus (27%) on cellular processes and signaling when compared to *S. coelicolor *(20%).

Besides these findings, only the genes for carbohydrate transport and -metabolism show another notable difference of more than 1% between *S. coelicolor *(13%) and *Actinoplanes *sp. SE50/110 (11%). Interestingly, 4% of the *Actinoplanes *sp. SE50/110 CDSs were found to be involved in secondary metabolite biosynthesis, a portion similar to the one found in the well-known producer *S. coelicolor *(5%) [52]. Taken together, these considerations lead to a new perception of the capabilities *Actinoplanes *sp. SE50/110 might offer, as for example, a new source of bioactive compounds. Furthermore and in contrast to *S. coelicolor*, the genome of *Actinoplanes *sp. SE50/110 hosts significantly more genes for signal transduction proteins. This might be one key to induce the expression of acarbose and novel secondary metabolite gene clusters by appropriately composed cultivation media following the OSMAC (one strain, many compounds) approach [53]. These considerations are in good accordance with empirical knowledge gathered through long lasting media optimizations [Bayer HealthCare AG, personal communication].

### Phylogenetic analysis of the *Actinoplanes *sp. SE50/110 16S rDNA reveals highest similarity to *Actinoplanes utahensis*

An unsupervised nucleotide BLAST [54] run of the 1509 bp long DNA sequence of the 16S rRNA gene from *Actinoplanes *sp. SE50/110 against the public non-redundant database (NCBI *nr/nt*) revealed high similarities to numerous species of the genera *Actinoplanes*, *Micromonospora *and *Salinispora*. Within the best 100 matches, the maximal DNA sequence identity was in the range of 100 - 96%. The coverage of the query sequence varied within this cohort between 100 - 97%. The hits with the highest similarity, based on the number of sequence substitutions were *A. utahensis *IMSNU 20044^T ^(17 substitutions, 3 gaps) and *A. utahensis *IFO 13244^T ^(16 substitutions, 3 gaps), both of which retrace to the type strain (^T^) *A. utahensis *ATCC 14539^T^, firstly described first by Couch in 1963 [2]. The third hit to *A. palleronii *IMSNU 2044^T ^differs from *Actinoplanes *sp. SE50/110 by 24 substitutions and 5 gaps.

**Figure 5:**
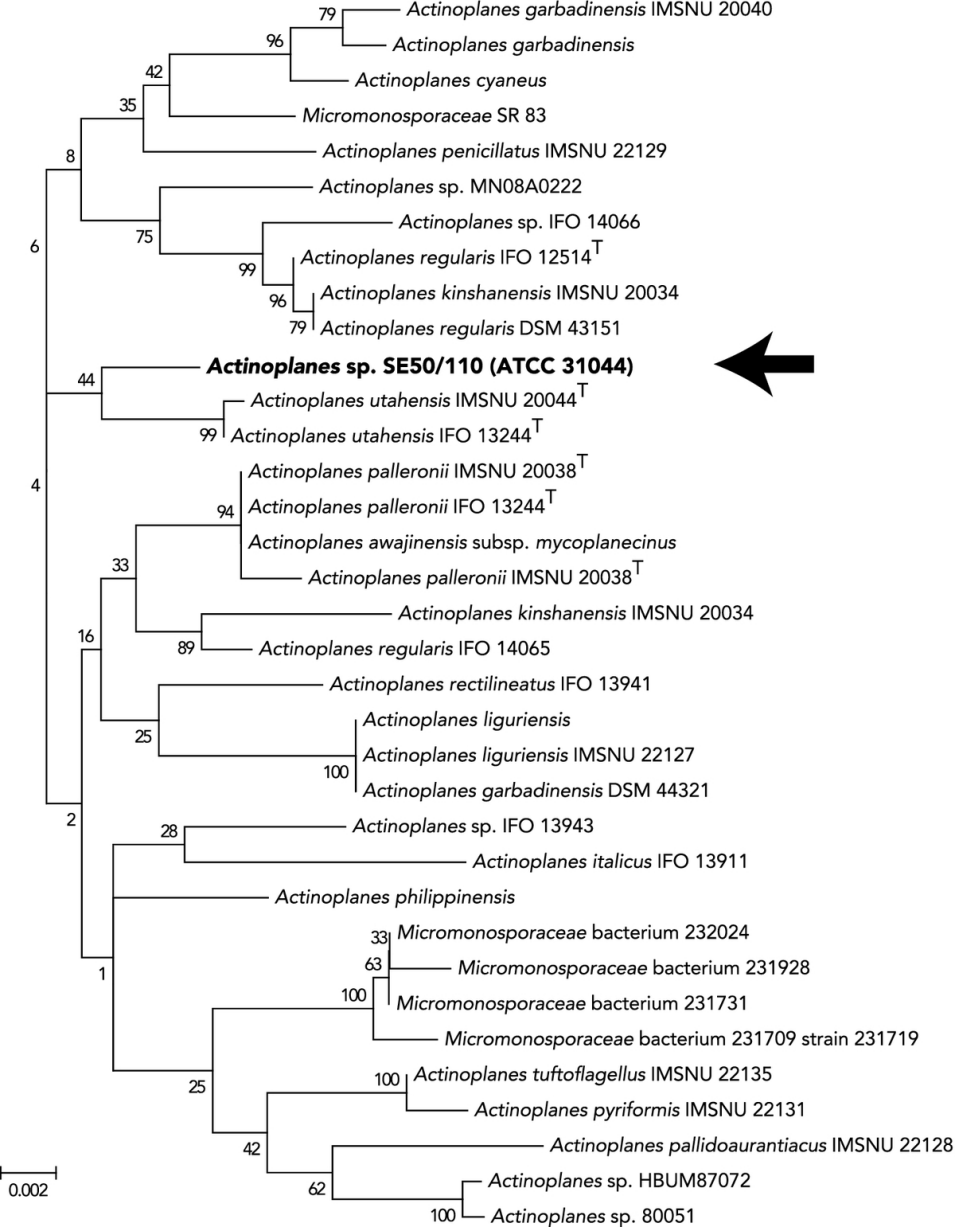
**Phylogenetic tree based on 16S rDNA from *Actinoplanes *sp. SE50/110 and the 34 most closely related species**. Shown is an excerpt of a phylogenetic tree build from the 100 best nucleotide BLAST hits for the *Actinoplanes *sp. SE50/110 16S rDNA. The shown subtree contains the 34 hits most closely related to *Actinoplanes *sp. SE50/110 (black arrow) with their evolutionary distances. The numbers on the branches represent confidence values in percent from a phylogenetic bootstrap test (1000 replications). The evolutionary history was inferred using the Neighbor-Joining method [56]. The bootstrap consensus tree inferred from 1000 replicates is taken to represent the evolutionary history of the taxa analyzed [57]. The tree is drawn to scale, with branch lengths in the same units as those of the evolutionary distances used to infer the phylogenetic tree. The evolutionary distances were computed using the Jukes-Cantor method [58] and are in the units of the number of base substitutions per site. The analysis involved 100 nucleotide sequences of which 35 are shown. Codon positions included were 1st + 2nd + 3 rd + Noncoding. All positions containing gaps and missing data were eliminated. There were a total of 1396 positions in the final dataset. Evolutionary analyses were conducted in MEGA5 [59]. Bar, 0.002 nucleotide substitutions per nucleotide position.

Based on the DNA sequences of the best 100 BLAST hits, a phylogenetic tree was derived. A detailed view on a subtree contains *Actinoplanes *sp. SE50/110 and 34 of the most closely related species (Figure 5). This subtree displays the derived phylogenetic distances between the analyzed strains, represented by their distance on the x-axis. From this analysis, it is evident that *A. utahensis *is the nearest species to *Actinoplanes *sp. SE50/110 currently publicly known, followed by *A. palleronii *and *A. awajiensis *subsp. *mycoplanecinus*. A second analysis using the latest version of the ribosomal database project [55] resulted in highly similar findings (data not shown). Interestingly, *A. utahensis *and *Actinoplanes *sp. SE50/110 form a subcluster within the *Actinoplanes *genus although the different isolates originate from far distant locations on different continents (Salt Lake City, USA, North America and Ruiru, Kenya, Africa). In addition, it is noteworthy that *Actinoplanes *sp. SE50/110 was renamed several times and in the early 1990s this strain was also classified as *A. utahensis *[49].

### Comparative genome analysis reveals 50% unique genes in the *Actinoplanes *sp. SE50/110 genome

**Figure 6:**
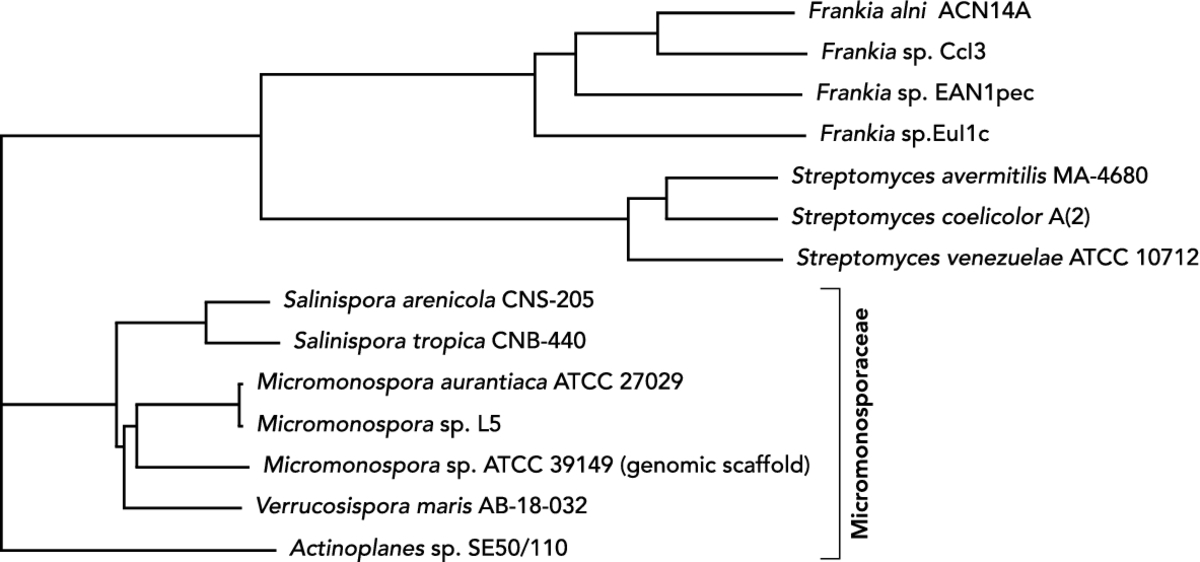
**Phylogenetic tree based on coding sequences (CDS) from *Actinoplanes *sp. SE50/110 and six species of the family Micromonosporaceae as well as *Streptomyces *and *Frankia *strains**. The tree was constructed using the software tool EDGAR [60] based on 605 core genome CDS from the species occurring in the analysis. The comparision shows all seven strains of the taxonomic family Micromonosporaceae sequenced and publicly available to date in relation to other well studied bacteria.

To date, seven full genome sequences belonging to the family Micromonosporaceae are publicly available. Using the comparative genomics tool EDGAR [60], a gene based, full genome phylogenetic analysis of these strains revealed a phylogeny comparable to the one inferred from 16S rRNA genes (Figure 6). For comparison, some industrially used *Streptomyces *and *Frankia *strains were also included in the analysis. As expected, each genus forms its own cluster. Interestingly, the genera *Micromonospora*, *Verrucosispora *and *Salinispora *are more closely related to each other than to *Actinoplanes*, whereas *Streptomyces *and *Frankia *are clearly distinct from the whole Micromonosporaceae family. Based on this analysis, the marine sediment isolate *Verrucosispora maris *AB-18-032 is the closest sequenced species to *Actinoplanes *sp. SE50/110 currently publicly known with 2,683 orthologous genes, a G + C content of 70.9% and a genome size of 6.67 MBases [61]. Comparative BLAST analysis of conserved orthologous genes of all sequenced Micromonosporaceae strains revealed prevalence for being located in the upper half of the genome, near the origin of replication (data not shown). The core genome analysis revealed a total of 1,670 genes common to all seven Micromonosporaceae strains, whereas the pan genome consists of 18,189 genes calculated by the EDGAR software [60]. An analysis of genes that exclusively occur in the *Actinoplanes *sp. SE50/110 genome revealed 4,122 singleton genes (49.8%).

The high quality genome sequence of *Actinoplanes *sp. SE50/110 corrects the previous sequence and annotation of the acarbose biosynthetic gene cluster

**Figure 7:**
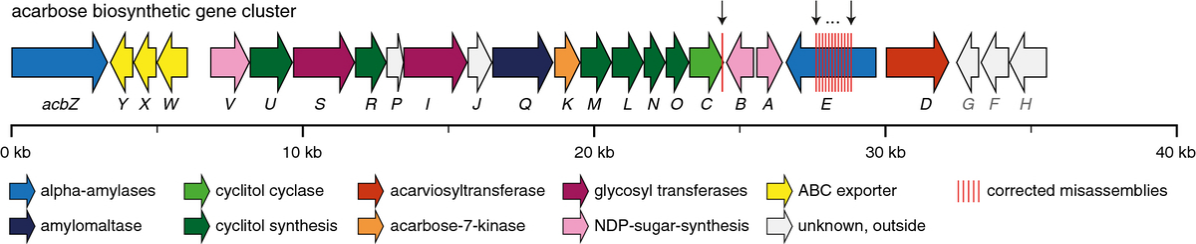
**The structure of the acarbose biosynthetic gene cluster from *Actinoplanes *sp. SE50/110**. Based on the whole genome sequence, several nucleotide corrections were found with respect to the previously sequenced reference sequence of the acarbose gene cluster [GenBank:Y18523.4]. The corrected sites in *acbC *and *acbE *are marked by arrows and red dashes.

The acarbose biosynthetic (*acb*) gene cluster sequencing was initiated [21] and successively expanded [17,24] by classical Sanger sequencing. Until now, this sequence was the longest (41,323 bp [GenBank:Y18523.4]) and best studied contiguous DNA fragment available from *Actinoplanes *sp. SE50/110. However, with the complete, high quality genome at hand, a total of 61 inconsistent sites were identified in the existing acarbose gene cluster sequence (Figure 7). Most notably, the deduced corrections affect the amino acid sequence of two genes, namely *acbC*, coding for the cytoplasmic 2-*epi*-5-*epi*-valiolone-synthase, and *acbE*, translating to a secreted long chain acarbose resistant α-amylase [17,24]. Because of two erroneous nucleotide insertions (c.1129_1130insG and c.1146_1147insC) in *acbC *(1197 bp), the resulting frameshift caused a premature stop codon to occur, shortening the actual gene sequence by 42 nucleotides. In *acbE *(3102 bp), the sequence differences are manifold, including mismatches, insertions and deletions, leading to multiple temporary frameshifts and single amino acid substitutions. Such differences occur in the mid part of the gene sequence, ranging from nucleotide position 1102 to 2247. These sequence corrections improved the similarity of the α-amylase domain to its catalytic domain family.

### Several genes of the acarbose gene cluster are also found in other locations of the *Actinoplanes *sp. SE50/110 genome sequence

It is known that the copy number of genes can have high impact on the efficiency of secondary metabolite production [19,62-64]. It is therefore worthwhile to study the genome wide occurrences of the genes encoded within the acarbose biosynthetic gene cluster, particularly with regard to import and export systems and the assessment of possible future knock-out experiments.

Our results show, that the *acb *gene cluster does not occur in more than one location within the *Actinoplanes *sp. SE50/110 genome. However, single genes and gene sets with equal functional annotation and amino acid sequence similarity to members of the *acb *cluster were found scattered throughout the genome by BLASTP analysis. Most notably, homologues to genes encoding the first, second and fourth step of the valienamine moiety synthesis of acarbose were found as a putative operon with moderate similarities of 52% (Acpl6250 to AcbC), 35% (Acpl6249 to AcbM) and 34% (Acpl6251 to AcbL). Furthermore, one homologue for each of the proteins AcbA (61% to Acpl3097) and AcbB (66% to Acpl3096) was identified. In the arcabose gene cluster, *acbA *and *acbB *are located adjacent to each other (Figure 7), and catalyze the first two sequential reactions needed for the formation of dTDP-4-keto-6-deoxy-D-glucose, another acrabose essential intermediate [21,28]. It is therefore interesting to note that the identified homologues to *acbA *and *acbB *were also found adjacent to each other in the context of a putative dTDP-rhamnose synthesis cluster (*acpl3095*-*acpl3098*). In *Mycobacterium smegmatis*, the orthologous dTDP-rhamnose biosynthetic gene cluster codes for mandatory proteins RmlABCD, involved in cell wall integrity and thus, cell survival [65]. Further genome analysis detected the ABC-transporters Acpl3214-Acpl3216 and Acpl5011-Acpl5013 with moderate similarity (28-49%) to the acarbose exporter complex AcbWXY. Both operons resemble the gene structure of *acbWXY *consisting of an ABC-type sugar transport ATP-binding protein and two ABC-type transport permease protein coding genes. Therefore, considering that ABC-transporters often present a promiscuous substrate-specificity, it is possible that these structurally similar transporters also be involved in acarbose transport. Acpl6399, a homologue with high sequence similarities to the alpha amylases AcbZ (65%) and AcbE (63%) was found encoded within the maltose importer operon *malEFG*. For the remaining *acb *genes only weak (*acbV*, *acbR*, *acbP*, *acbJ*, *acbQ*, *acbK *and *acbN*) or no similarities (*acbU*, *acbS*, *acbI *and *acbO*) were found outside of the *acb *cluster by BLASTP searches using an e-value threshold of e^-10^.

In contrast to previous findings [27], the extracellular binding protein AcbH, encoded within the *acbGFH *operon (Figure 7), was recently shown to exhibit high affinity to galactose instead of acarbose or its homologues [22]. This implicates that *acbGFH *does not directly belong to the acarbose cluster as was proposed by the carbophore hypothesis, which indicates that acarbose or acarbose homologues can be reused by the producer [17,66]. In order to search the *Actinoplanes *sp. SE50/110 genome for a new acarbose importer candidate, the *gacGFH *operon of a second acarbose gene cluster identified in *Streptomyces glaucescens *GLA.O has been used as query [67]. GacH was recently shown to recognize longer acarbose homologues but exhibits only low affinity to acarbose [68]. However, the search revealed rather weak similarities towards the best hit operon *acpl5404*-*acpl5406 *with GacH showing 26% identity to its homologue Acpl5404. A consecutive search of the extracellular maltose binding protein MalE from *Salmonella typhimurium*, which has been shown to exhibit high affinity to acarbose [68], revealed 32% identity to its MalE homologue in *Actinoplanes *sp. SE50/110. Despite the low sequence similarities, these findings suggest that acarbose or its homologues are either imported by one or both of the above mentioned importers, or that the extracellular binding protein exhibits a distinct amino acid sequence in *Actinoplanes *sp. SE50/110 and can therefore not be identified by sequence comparison alone.

### The *Actinoplanes *sp. SE50/110 genome hosts an integrative and conjugative element that also exists in multiple copies as an extrachromosomal element

**Figure 8:**
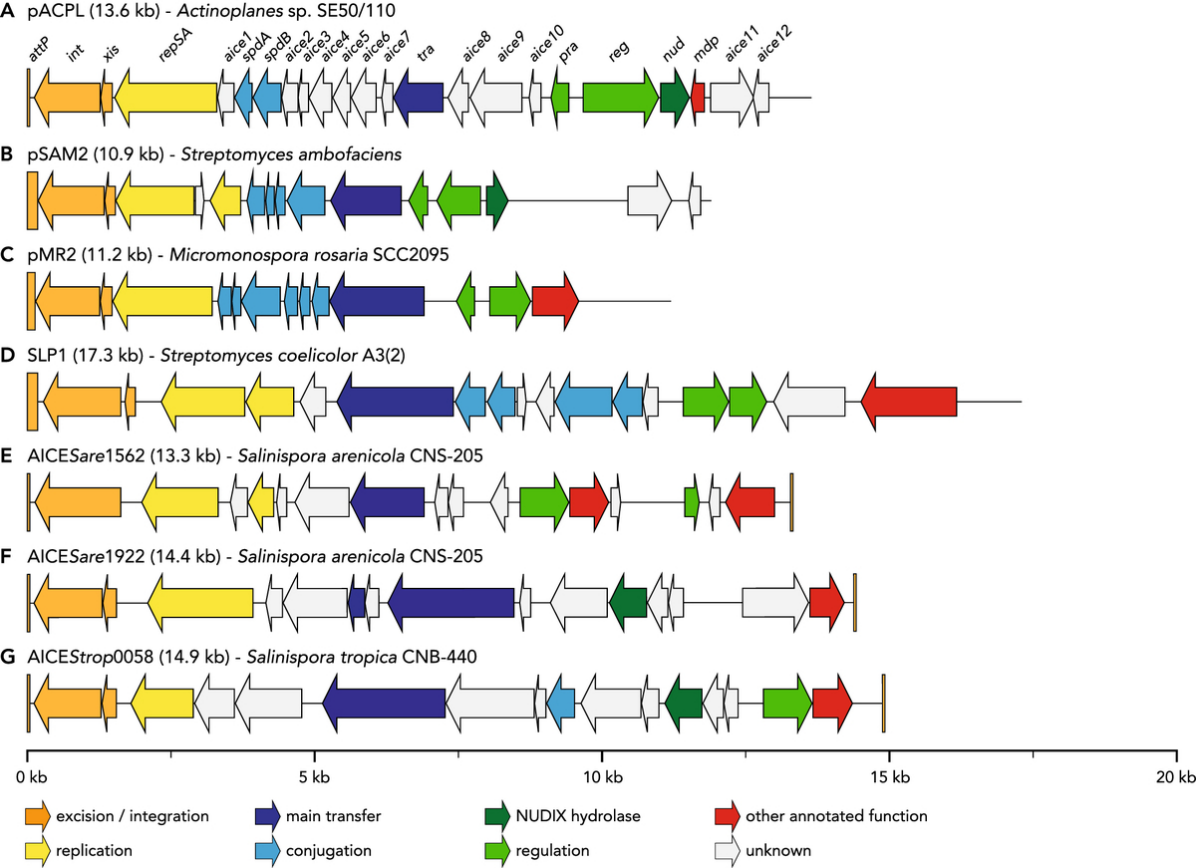
**Structural organization of the newly identified actinomycete integrative and conjugative element (AICE) pACPL from *Actinoplanes *sp. SE50/110 in comparison with other AICEs from closely related species**. (**A**) pACPL (13.6 kb), the first AICE found in the *Actinoplanes *genus from *Actinoplanes *sp. SE50/110; (**B**) pSAM2 (10.9 kb) from *Streptomyces ambofaciens*; (**C**) pMR2 (11.2 kb) from *Micromonospora rosaria *SCC2095; (**D**) SLP1 (17.3 kb) from *Streptomyces coelicolor *A3(2); (**E, F**) AICE*Sare*1562 (13.3 kb) and AICE*Sare*1922 (14.4 kb) from *Salinispora arenicola *CNS-205; (**G**) AICE*Strop*0058 (14.9 kb) from *Salinispora tropica *CNB-440. B-G adapted from [69].

Actinomycete integrative and conjugative elements (AICEs) are a class of mobile genetic elements possessing a highly conserved structural organization with functional modules for excision/integration, replication, conjugative transfer and regulation [69]. Being able to replicate autonomously, they are also said to mediate the acquisition of additional modules, encoding functions such as resistance and metabolic traits, which confer a selective advantage to the host under certain environmental conditions [70]. Interestingly, a similar AICE, designated pACPL, was identified in the complete genome sequence of *Actinoplanes *sp. SE50/110 (Figure 8). Its size of 13.6 kb and the structural gene organization are in good accordance with other known AICEs of closely related species like *Micromonospora rosario*, *Salinispora tropica *or *Streptomyces coelicolor *(Figure 8).

Most known AICEs subsist in their host genome by integration in the 3' end of a tRNA gene by site-specific recombination between two short identical sequences (*att *identity segments) within the attachment sites located on the genome (*attB*) and the AICE (*attP*), respectively [69]. In pACPL, the *att *identity segments are 43 nt in size and *attB *overlaps the 3' end of a proline tRNA gene. Moreover, the identity segment in *attP *is flanked by two 21 nt repeats containing two mismatches: GTCACCCAGTTAGT(T/C)AC(C/T)CAG. These exhibit high similarities to the arm-type sites identified in the AICE pSAM2 from *Strepomyces ambofaciens*. For pSAM2 it was shown that the integrase binds to these repeats and that they are essential for efficient recombination [71].

pACPL hosts 22 putative protein coding sequences (Figure 8). The integrase, excisionase and replication genes *int*, *xis *and *repSA *are located directly downstream of *attP *and show high sequence similarity to numerous homologues from closely related species. The putative main transferase gene *tra *contains the sequence of a FtsK-SpoIIIE domain found in all AICEs and *Streptomyces *transferase genes [69]. SpdA and SpdB show weak similarity to spread proteins from *Frankia *sp. CcI3 and *M. rosaria *where they are involved in the intramycelial spread of AICEs [72,73]. The putative regulatory protein Pra was first described in pSAM2 as an AICE replication activator [74]. On pACPL, it exhibits high similarity to an uncharacterized homologue from *Micromonospora aurantica *ATCC 27029. A second regulatory gene *reg *shows high similarities to transcriptional regulators of various *Streptomyces *strains whereas the downstream gene *nud *exhibits 72% similarity to the amino acid sequence of a NUDIX hydrolase from *Streptomyces *sp. AA4. In contrast, *mdp *codes for a metal dependent phosphohydrolase also found in various *Frankia *and *Streptomyces *strains.

Homologues to the remaining genes are poorly characterized and largely hypothetical in public databases although *aice4 *is also found in various related species and shows akin to *aice1*, *aice2*, *aice5*, *aice6*, and *aice9*, high similarity to homologues from *M. aurantiaca*. Interestingly, homologues to *aice1 *and *aice2 *were only found in *M. aurantiaca*, whereas *aice3*, *aice7*, *aice8*, *aice10*, *aice11*, and *aice12*, seem to solely exist in *Actinoplanes *sp. SE50/110.

Based upon read-coverage observations of the AICE containing genomic region, an approximately twelve-fold overrepresentation of the AICE coding DNA sequences has been revealed (Figure 1). As only one copy of the AICE was found to be integrated in the genome, it was concluded that approximately eleven copies of the element might exist as circular, extrachromosomal versions in a typical *Actinoplanes *sp. SE50/110 cell. However, the number of extrachromosomal copies per cell might be even higher, as it is possible that a proportion of the AICEs was lost during DNA isolation. It should also be noted that the rather low G + C content of the AICE (65.56%) might have introduced a similar amplification bias during the library preparation as discussed above for the *rrn *operons. Nevertheless, these findings are of great interest, as they demonstrate the first native functional AICE for *Actinoplanes *spp. in general and imply the possibility of future genetic access to *Actinoplanes *sp. SE50/110 in order to perform targeted genetic modifications as done before for e.g. *Micromonospora *spp. [75]. The newly identified AICE may also improve previous efforts in the analysis of heterologous promoters for the overexpression of the lipopeptide antibiotic friulimicin in *Actinoplanes friuliensis *[76].

### Four putative antibiotic production gene clusters were found in the Actinoplanes sp. SE50/110 genome sequence

Bioactive compounds synthetized through secondary metabolite gene clusters are a rich source for pharmacologically relevant products like antibiotics, immunosuppressants or antineoplastics [77,78]. Besides aminoglycosides, the majority of these metabolites are built up in a modular fashion by using non-ribosomal peptide synthetases (NRPS) and/or polyketide synthases (PKS) as enzyme templates (for a recent review see [79]). Briefly, the nascent product is built up by sequential addition of a new element at each module it traverses. The complete sequence of modules may reside on one gene or spread across multiple genes in which the order of action of each gene product is determined by specific linker sequences present at proteins' N- and C-terminal ends [78,80].

For NRPSs, a minimal module typically consists of at least three catalytic domains, namely the andenylation (A) domain for specific amino acid activation, the thiolation (T) domain, also called peptidyl carrier protein (PCP) for covalent binding and transfer and the condensation (C) domain for incorporation into the peptide chain [78]. In addition, domains for epimerization (E), methylation (M) and other modifications may reside within a module. Oftentimes a thioesterase domain (Te) is located at the C-terminal end of the final module, responsible for e.g. cyclization and release of the non-ribosomal peptide from the NRPS [81].

In case of the PKSs, an acyltransferase (AT) coordinates the loading of a carboxylic acid and promotes its attachment on the acyl carrier protein (ACP) where chain elongation takes place by a β-kethoacyl synthase (KS) mediated condensation reaction [79]. Additionally, most PKSs reduce the elongated ketide chain at accessory β-kethoacyl reductase (KR), dehydratase (DH), methyltransferase (MT) or enoylreductase (ER) domains before a final thioesterase (TE) domain mediates release of the polyketide [82].

Modular NRPS and PKS enzymes strictly depend on the activation of the respective carrier protein domains (PCP and ACP), which must be converted from their inactive apo-forms to cofactor-bearing holo-forms by a specific phosphopantetheinyl transferase (PPTase) [83]. The genome of *Actinoplanes *sp. SE50/110 hosts three such enzyme encoded by the genes *acpl842*, *acpl996*, and *acpl6917*.

**Figure 9:**
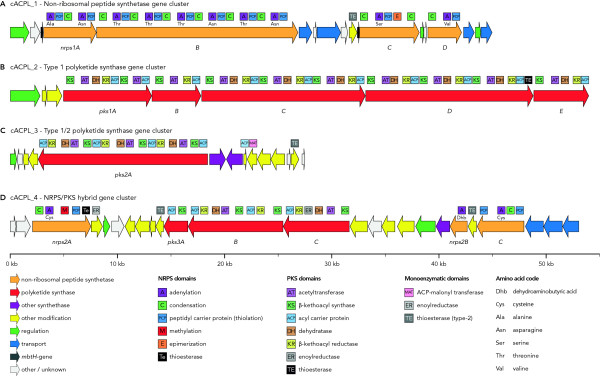
**The gene organization of the four putative secondary metabolite gene clusters found in the *Actinoplanes *sp. SE50/110 genome**. (**A**) Non-ribosomal peptide synthetase (NRPS) cluster showing high structural and sequential similarity to the SMC14 gene cluster identified on the pSCL4 megaplasmid from *Streptomyces clavuligerus *ATCC 27064. (**B**) Large polyketide synthase (PKS) gene cluster exhibiting 62-66% similarity to PKSs from various *Streptomyces *strains. (**C**) A single PKS gene with various accessory genes showing some structural similarity to a yet uncharacterized PKS gene cluster of *Salinispora tropica *CNB-440. (**D**) Putative hybrid NRPS/PKS gene cluster with NRPS genes showing high similarity (63-76%) to genes from an uncharacterized cluster of *Streptomyces venezuelae *ATCC 10712 whereas the PKS genes exhibit highest similarity (63-66%) to genes scattered in the *Methylosinus trichosporium *OB3b genome.

In *Actinoplanes *sp. SE50/110, one NRPS (cACPL_1), two PKS (cACPL_2 & cACPL_3) and a hybrid NRPS/PKS cluster (cACPL_4) were found by gene annotation and subsequent detailed analysis using the antiSMASH pipeline [84]. The first of the identified gene clusters (cACPL_1) contains four NRPS genes (Figure 9A), hosting a total of ten adenylation (A), thiolation (T) and condensation (C) domains, potentially making up 10 modules. Thereof, three modules are formed by intergentic domains, which suggests a specific interaction of the four putative NRPS enzymes in the order *nrps1A*-*B*-*D*-*C*. Such interaction order is the only one that leads to the assembly of all domains into 10 complete modules - 9 minimal modules (A-T-C) and one module containing an additional epimerization domain. These considerations were corroborated by matching linker sequences, named short communication-mediating (COM) domains [78], found at the C-terminal part of NRPS1D and the N-terminal end of NRPS1C. Furthermore, this cluster shows high structural and sequential similarity to the SMC14 gene cluster identified on the pSCL4 megaplasmid from *Streptomyces clavuligerus *ATCC 27064 [85]. However, in SMC14 a homolog to *nrps1D *is missing, which leads to the speculation that *nrps1D *was subsequently added to the cluster as an additional building block. In fact, leaving *nrps1D *out of the assembly line would theoretically still result in a complete enzyme complex built from 9 instead of 10 modules. Based on the antiSMASH prediction, the amino acid backbone of the final product is likely to be composed of the sequence: Ala-Asn-Thr-Thr-Thr-Asn-Thr-Asn-Val-Ser (Figure 9A). Besides the NRPSs, the cluster also contains multiple genes involved in regulation and transportation as well as two *mbtH*-like genes, known to facilitate secondary metabolite synthesis. In this regard, it is noteworthy that the occurrence of two *mbtH *genes in a secondary metabolite gene cluster is exceptional in that it has only been found once before in the teicoplanin biosynthesis gene cluster of *Actinoplanes teichomyceticus *[86].

The type-1 PKS-cluster cACPL_2 (Figure 9B) hosts 5 genes putatively involved in the synthesis of an unknown polyketide. The sum of the PKS coding regions adds up to a size of ~49 kb whereas all encoded PKSs exhibit 62-66% similarity to PKSs from various *Streptomyces *strains. However unlike the NRPS-cluster, no cluster structurally similar to cACPL_2 was found in public databases. Analysis of the domain and module architecture revealed a total of 10 elongation modules (KS-AT-[DH-ER-KR]-ACP) including 9 β-kethoacyl reductase (KR) and 8 dehydratase (DH) domains as well as a termination module (TE). However, an initial loading module (AT-ACP) could not be identified in the proximity of the cluster. To elucidate the most likely build order of the polyketide, the N- and C-terminal linker sequences were matched against each other using the software SBSPKS [87] and antiSMASH. Remarkably, both programs independently predicted the same gene order: *pks1E*-*C*-*B*-*A*-*D*.

Just 15 kb downstream of cACPL_2, a second gene cluster (cACPL_3) containing a long PKS gene with various accessory protein coding sequences could be identified (Figure 9C). It shows some structural similarity to a yet uncharacterized PKS gene cluster of *Salinispora tropica *CNB-440 (genes *Strop_2768 *- *Strop_2777*). Besides the 3 elongation modules identified on *pks2A*, no other modular type 1 PKS genes were found in the proximity of the cluster. However, genes downstream of pks2A are likely to be involved in the synthesis and modification of the polyketide, coding for an acyl carrier protein (ACP), an ACP malonyl transferase (MAT), a lysine aminomutase, an aspartate transferase and a type 2 thioestrase. Especially type 2 thioestrases are often found in PKS clusters [88] like e.g., in the gramicidin S biosynthesis operon [89]. The presence of discrete ACP, MAT and two additional acetyl CoA synthetase-like enzymes is also typical for type 2 PKS systems [90] although no ketoacyl-synthase (KS_α_) and chain length factor (KS_β_) was found in this cluster [91].

Another 58 kb downstream of cACPL_3 a fourth secondary metabolite cluster (cACPL_4) was located (Figure 9D). It hosts both 3 NRPS and 3 PKS genes and may therefore synthesize a hybrid product as previously reported for bleomycin from *Streptomyces verticillus *[92], pristinamycin IIB from *Streptomyces pristinaespiralis *[93] and others [94]. N- and C-terminal sequence analysis of the two cluster types revealed the gene orders *nrps2B*-*C*-*A *and *pks3A*-*B*-*C *as most likely. The prediction of the peptide backbone of the NRPS cluster resulted in the putative product dehydroaminobutyric acid (Dhb)-Cys-Cys. One could speculate that the PKSs are used prior to the NRPSs as *nrps2A *comes with a termination module (Te). However, two additional monomeric thioesterase (TE) and one enoylreductase (ER) domain containing genes do also belong to the cluster and may be involved in the termination and modification of the product. Notably, all three NRPS genes show high similarity (63-76%) to genes from an uncharacterized cluster of *Streptomyces venezuelae *ATCC 10712 whereas the PKS genes exhibit highest similarity (63-66%) to genes scattered in the *Methylosinus trichosporium *OB3b genome.

The four newly discovered secondary metabolite gene clusters broaden our knowledge of actinomycete NRPS and PKS biosynthesis clusters and represent just the tip of the iceberg of the manifold biosynthetical capabilities - apart from the well-known acarbose production - that *Actinoplanes *sp. SE50/110 houses. It remains to be determined if all presented clusters are involved in industrially rewarding bioactive compound synthesis and how these clusters are regulated, because none of these metabolites were identified and isolated so far. These new gene clusters may also be used in conjunction with well-studied antibiotic operons, in order to synthesize completely new substances, as recently performed [95,96].

## Conclusions

The establishment of the complete genome sequence of the acarbose producer *Actinoplanes *sp. SE50/110 is an important achievement on the way towards rational optimization of the acarbose production through targeted genetic engineering. In this process, the identified AICE may serve as a vector for future transformation of *Actinoplanes *spp. Furthermore, our work provides the first sequenced genome of the genus *Actinoplanes*, which will serve as the reference for future genome analysis and sequencing projects in this field. By providing novel insights into the enzymatic equipment of *Actinoplanes *sp. SE50/110, we identified previously unknown NRPS/PKS gene clusters, potentially encoding new antibiotics and other bioactive compounds that might be of pharmacologic interest.

With the complete genome sequence at hand, we propose to conduct future transcriptome studies on *Actinoplanes *sp. SE50/110 in order to analyze differential gene expression in cultivation media that promote and repress acarbose production, respectively. Results will help to identify potential target genes for later genetic manipulations with the aim of increasing acarbose yields.

## Methods

### Cultivation of the *Actinoplanes *sp. SE50/110 strain

In order to isolate DNA, the *Actinoplanes *sp. SE50/110 strain was cultivated in a two-step shake flask system. Besides inorganic salts the medium contained starch hydrolysate as carbon source and yeast extract as nitrogen source. Pre culture and main culture were incubated for 3 and 4 days, respectively, on a rotary shaker at 28°C. Then the biomass was collected by centrifugation.

### Preparation of genomic DNA

The preparation of genomic DNA of the *Actinoplanes *sp. SE50/110 strain was performed as previously published [30].

### High throughput sequencing and automated assembly of the *Actinoplanes *sp. SE50/110 genome

The high throughput pyrosequencing has been carried out on a Genome Sequencer FLX system (454 Life Sciences). The subsequent assembly of the generated reads was performed using the Newbler assembly software, version 2.0.00.22 (454 Life Sciences). Details of the sequencing and assembly procedures have been described previously [30].

### Construction of a fosmid library for the *Actinoplanes *sp. SE50/110 genome finishing

The fosmid library construction for *Actinoplanes *sp. SE50/110 with an average inset size of 40 kb has been carried out on isolated genomic DNA by IIT Biotech GmbH (Universitätsstrasse 25, 33615 Bielefeld, Germany). For construction in *Escherichia coli *EPI300 cells, the CopyControl™ Cloning System (EPICENTRE Biotechnologies, 726 Post Road, Madison, WI 53713, USA) has been used. The kit was obtained from Biozym Scientific GmbH (Steinbrinksweg 27, 31840 Hessisch Oldendorf, Germany).

### Terminal insert sequencing of the *Actinoplanes *sp. SE50/110 fosmid library

The fosmid library terminal insert sequencing was carried out with capillary sequencing technique on a 3730xl DNA-Analyzer (Applied Biosystems) by IIT Biotech GmbH. The resulting chromatogram files were base called using the phred software [97,98] and stored in FASTA format. Both files were later used for gap closure and quality assessment.

### Finishing of the *Actinoplanes *sp. SE50/110 genome sequence by manual assembly

In order to close remaining gaps between contiguous sequences (contigs) still present after the automated assembly, the visual assembly software package Consed [40,99] was utilized. Within the graphical user interface, fosmid walking primer and genome PCR primer pairs were selected at the ends of contiguous contigs. These were used to amplify desired sequences from fosmids or genomic DNA in order to bridge the gaps between contiguous contigs.

After the DNA sequence of these amplicons had been determined, manual assembly of all applicable reads was performed with the aid of different Consed program features. In cases where the length or quality of one read was not sufficient to span the gap, multiple rounds of primer selection, amplicon generation, amplicon sequencing and manual assembly were performed.

### Prediction of open reading frames on the *Actinoplanes *sp. SE50/110 genome sequence

The potential genes were identified by a series of programs which are all part of the GenDB annotation pipeline [44]. For the automated identification of open reading frames (ORFs) the prokaryotic gene finders Prodigal [42] and GISMO [43] were primarily used. In order to optimize results and allow for easy manual curation, further intrinsic, extrinsic and combined methods were applied by means of the Reganor software [100,101] which utilizes the popular gene prediction tools Glimmer [102] and CRITICA [103].

### Functional annotation of the identified open reading frames of the *Actinoplanes *sp. SE50/110 genome

The identified open reading frames were analyzed through a variety of different software packages in order to draw conclusions from their DNA- and/or amino acid-sequences regarding their potential function. Besides functional predictions, further characteristics and structural features have also been calculated.

Similarity-based searches were applied to identify conserved sequences by means of comparison to public and/or proprietary nucleotide- and protein-databases. If a significant sequence similarity was found throughout the major section of a gene, it was concluded that the gene should have a similar function in *Actinoplanes *sp. SE50/110. The similarity-based methods, which were used to annotate the list of ORFs are termed BLASTP [104] and RPS-BLAST [105].

Enzymatic classification has been performed on the basis of *enzyme commission *(EC) numbers [46,106]. These were primarily derived from the PRIAM database [107] using the PRIAM_Search utility on the latest version (May 2011) of the database. For genes with no PRIAM hit, secondary EC-number annotations were derived from searches against the Kyoto Encyclopedia of Genes and Genomes (KEGG) database [108,109]. For further functional gene annotation, the *cluster of orthologous groups of proteins *(COG) classification system has been applied [45,51] using the latest version (March 2003) of the database [110].

To identify potential transmembrane proteins, the software TMHMM [111,112] has been utilized.

The software SignalP [113-115] was used to predict the secretion capability of the identified proteins. This is done by means of Hidden Markov Models and neural networks, searching for the appearance and position of potential signal peptide cleavage sites within the amino acid sequence. The resulting score can be interpreted as a probability measure for the secretion of the translated protein. SignalP retrieves only those proteins which are secreted by the classical signal-peptide-bound mechanisms.

In order to identify further *Actinoplanes *sp. SE50/110 proteins which are not secreted via the classical way, the software SecretomeP has been applied [116]. The underlying neural network has been trained with secreted proteins, known to lack signal peptides despite their occurrence in the exoproteome. The final secretion capability of the translated genes was derived by the combined results of SignalP and SecretomeP predictions.

To reveal polycistronic transcriptional units, proprietary software has been developed which predicts jointly transcribed genes by their orientation and proximity to neighboring genes (adopted from [117]). In light of these predictions, operon structures can be estimated and based upon them further sequence regions can be derived with high probability of contained promoter and operator elements.

The software DNA mfold [118] has been used to calculate hybridization energies for the DNA sequences in order to identify secondary structures such as transcriptional terminators which indicate operon and gene ends, respectively. The involved algorithm computes the most stable secondary structure of the input sequence by striving to the lowest level in terms of Gibbs free energy (ΔG), a measure for the energy which is released by the formation of hydrogen bonds between the hybridizing base pairs.

### Phylogenetic analyses

The 16S rDNA-based phylogenetic analysis was performed on DNA sequences retrieved by public BLAST [54] searches against the 16S rDNA of *Actinoplanes *sp. SE50/110. From the best hits, a multiple sequence alignment was built by applying the MUSCLE program [119,120] before deriving the phylogeny thereof by the MEGA 5 software [59] using the neighbor-joining algorithm [56] with Jukes-Cantor model [58].

Genome based phylogenetic analysis of *Actinoplanes *sp. SE50/110 in relation to other closely related species was performed with the comparative genomics tool EDGAR [60]. Briefly, the core genome of all selected strains was calculated and based thereon, phylogenetic distances were calculated from multiple sequence alignments. Then, phylogenetic trees were generated from concatenated core gene alignments.

## Competing interests

The authors declare that they have no competing interests.

## Authors' contributions

AP, KS and JK designed the study. KS was responsible for the cultivation and DNA isolation. RS and CR performed the library preparation and high-throughput sequencing. AP, UFW, RS and CR critically revised the manuscript. AP, UFW, JS and AK provided important intellectual contributions. PS carried out all work not contributed for by other authors and wrote the manuscript. All authors read and approved the manuscript.
